# Effects of Pore Size on Fatigue Deformation Mechanism of Open-Cell Copper Foam at Low Stress Amplitude

**DOI:** 10.3390/ma11091639

**Published:** 2018-09-06

**Authors:** Jian Chen, Shuowei Dai, Cong Li, Wei Li, Yanjie Ren

**Affiliations:** 1 School of Energy and Power Engineering, Changsha University of Science & Technology, Changsha 410014, China; chenjian513@csust.edu.cn (J.C.); shuoweidai@126.com (S.D.); lwzzgjajie@126.com (W.L.); yjren@csust.edu.cn (Y.R.); 2 Key Laboratory of Energy Efficiency and Clean Utilization, Education Department of Hunan Province, Changsha University of Science & Technology, Changsha 410014, China; 3 Guangxi Key Laboratory of Electrochemical Energy Materials, Guangxi University, Nanning 530004, China

**Keywords:** open-cell copper foam, pore size, failure mechanism

## Abstract

Axial compression-compression fatigue experiments on open-cell copper foams with different pore size were investigated in this paper. The effects of the strain amplitude on the fatigue properties were studied and found that there is an exponential relationship between the fatigue life and strain amplitude. The experimental results indicate that a smaller pore size is related to a lower fatigue life. The microstructures of failed copper foam tested at low stress amplitude were observed by optical microscope and scanning electron microscopy (SEM), suggests that different pore size related to different fatigue behavior. The fatigue failure mechanism of the open-cell copper foams were compared by experimental research.

## 1. Introduction

Metal foams have been extensively applied in many industrial devices such as the electrodes of lithium-ion battery and heat exchangers due to its light weight and excellent thermal conductivity [1,2,3]. Current collector is one of the most important part of lithium-ion battery which acts as the electron transmission channel between internal and external circuit. The performance of battery is largely determined by the current collector [4,5,6,7]. Until now, in commercial lithium-ion batteries, most of the negative current collector is made by electrolytic copper foil. However, the volume change of the anode materials during charge/discharge cycles can easily result in the separation of negative charged materials and copper foil. To obtain a better property of lithium–ion battery, open-cell foam copper has been proposed for the using as a current collector material for its porous cellular structure and excellent performance [8,9].

Until now, recent studies on the fatigue property and failure mechanism of foam metal have focused primarily on aluminum foam [10,11]. Zettl studied the high-cycle fatigue properties of aluminum foam, the results showed that fatigue cracks initiate from the thinnest struts and terminate near the pore node [12]. When the initial defect density and the pore size are decreased or the pore size is more uniform, the cyclic performance of aluminum foam is improved [13]. Ingrahamat investigated the low-cycle fatigue behavior and failure model of aluminum foam through testing and digital image analysis. It was found that the compaction-assisted crack propagation was an important driving force for the failure process and those cracks further extended to the side of the hysteresis loop [14]. 

However, the mechanical properties of copper foam are seldom investigated [15,16,17]. El-Hadek studied the compression properties of copper foams and found that the elastic modulus was consistent with the Mori-Tanaka predictions and that Young's modulus decreased with decreasing porosity [18]. In our previous research, we studied the compression properties and deformation failure mechanism of open-cell copper foam and confirmed that the deformation of copper foam is caused predominantly by strut bending and the failure mechanism of the open-cell copper foam is layer-by-layer collapse [19,20,21]. Pore size, as an important parameter of copper foam, also plays a significant role in mechanical properties which can also affect the fatigue life. With an aim to obtain more information about the mechanical property of high-porosity copper foam, in present study, the fatigue performance of open-cell copper foam with different pore size was investigated.

## 2. Experimental

### 2.1. Material

The material used for the experiments is a commercial product, and it was made by Suzhou Jia Long De metal foam Co. Ltd. (Suzhou, China) All of the open-cell copper foam was prepared by electro-deposition. The matrix is polyurethane foam with PPI (pores per inch, 1 in = 25.4 mm) values of 5, 20 and 40 (The value of PPI is provided directly by the Suzhou Jia Long De metal foam Co. Ltd. Suzhou, China) After the chemical treatment, a layer of conductive copper film was deposited on the surface of the polyurethane, and then the plating treatment was conducted. Finally, the polyurethane matrix was removed in an atmosphere furnace, open-cell copper foam with a high porosity and a hollow structure was produced. Samples with different pore size were cut into 24 mm × 30 mm cylindrical specimens using Wire cut Electrical Discharge Machining (WEDM). The samples were weighed by electronic weighing, and the porosity (*P*) and the relative density ($\rho_{R}$) of the samples was calculated according to Equations (1) and (2) [22].

(1) $$P=1-\frac{4m}{\pi \rho h\Phi^{2}}\;$$

(2) $$\rho_{R}=1-P\;$$

where *m*, *h*, Φ are the mass, height and diameter of the sample, respectively. *ρ* is the density of skeletal density. The characteristic parameters of the open-cell copper foam used in the experiments are shown in Table 1. Three types of copper foams were tested.

### 2.2. Experiment Methods

Before the experiment, in order to remove the surface oxidation, samples were placed in a H_2_ atmosphere at a temperature of 700 °C for 4 h. After cooling in the heating furnace (Changsha Kexin Co., Ltd., Changsha, China), the samples were placed in an acetone (Hengyang Kaixin Chemical Reagent Co., Ltd., Hengyang, China) solution and ultrasonically cleaned for 15 min to eliminate the effect of carbon residue.

The fatigue performance of the copper foams was evaluated using a compression/compression testing machine (IBTC 2000: made by Care Test & Control System (China) Co., Ltd., Tianjin, China). The maximum load of the fatigue machine is 2 KN, and the measuring accuracy is 10^−4^. Both stress and strain were recorded by a computer. The cyclic stress applied was sinusoidal at a frequency of 1 Hz. According to the quasi-static compression result in our previous study, the yield strength of these copper foam was determined to be around 1 MPa. Several different stress points with the values lower than the yield strength are selected as stress amplitudes. In these tests, the stress amplitude varied from 0.348 MPa to 0.448 MPa [19]. The corresponding loads are calculated according to the relationship between load and stress. For each condition, 3 samples were tested. The ratio of minimum stress to maximum stress (R) was kept at a constant value of 0.1. The experimental parameters of the compression-compression fatigue test were shown in Table 2. The fatigue failure criterion adopted in the experiment is that the strain reaches 30% or the macroscopic failure is achieved.

The macroscopic deformation of specimen was observed continuously by NSDS2100 non-contact video extensometer (Care Test & Control System (China) Co., Ltd., Tianjin, China). The microstructure of specimens after deformation were examined by 200MAT optical microscope (Carle Zeiss AG, Weimar, Germany) and Quanta FEG 250 scanning electron microscopy (SEM) (FEI, Hillsboro, OR, USA) system. 

## 3. Results and Discussion

### 3.1. Fatigue Properties

The fatigue life curve can be described as Equation (3) which in the form of power function involve two parameters:

(3) $$\sigma_{a}=\Delta \sigma /2=a{\left(N\right)}^{b}=\left(\sigma_{f}-\sigma_{m}\right){\left(2N\right)}^{b}\;$$

where *σ_a_* is stress amplitude of fatigue load which can also be described by Δ*σ*/2, *N* is the cycle fatigue life, *σ_f_* is fatigue strength coefficient, *σ_m_* is the mean stress and *a*, *b* are constants. By using R-square number, the experimental data can be fitted by the least square method, as the value of R-square usually varies from 0 to 1 [23]. A larger number of R-square accounts for a better fitting consequence. Hossain also uses the power law equations to describe the fatigue behaviour for foam materials [24].

**Figure 1:**
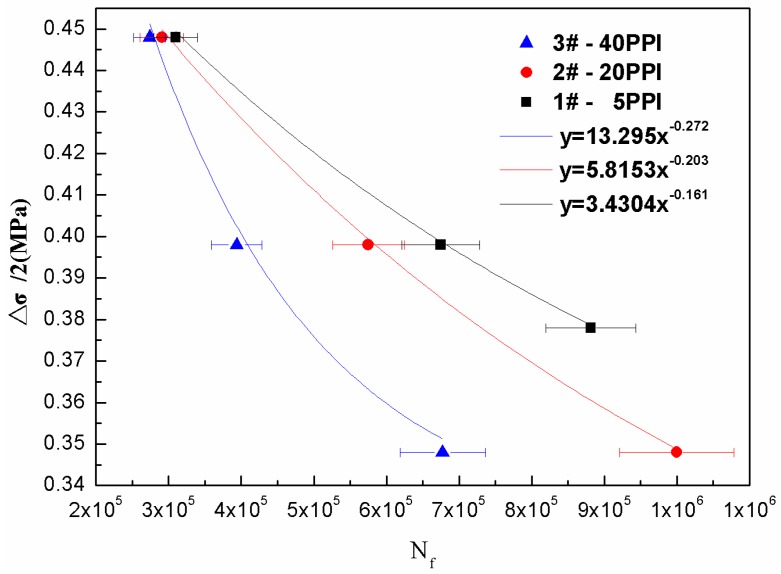
Fatigue life curve of the compression-compression fatigue test.

Figure 1 is the fatigue test result of open-cell copper foam with different pore size, showing the number of cycles to failure. It could be observed that the simulated data were in a good agreement with the experiment data. It is obvious that a larger stress amplitude result in a lower cycle number. Samples with larger pore size (lower PPI) related to a higher compression strength at low stress amplitude. It should be noted that another experiment with a stress amplitude of 0.378 MPa was carried out on 5PPI copper foam samples, because no failure was observed when the stress amplitude is set as 0.348 MPa. As shown in Figure 1, the fatigue life were similar when samples with different pore size were tested at the stress amplitude of 0.448 MPa. However, a larger pore size related to a longer fatigue life with the decreasing stress amplitude. In conclusion, the fatigue life of the open-cell copper foam at stress amplitude of 0.348 MPa followed the order: 5PPI > 20PPI > 40PPI. 

**Figure 2:**
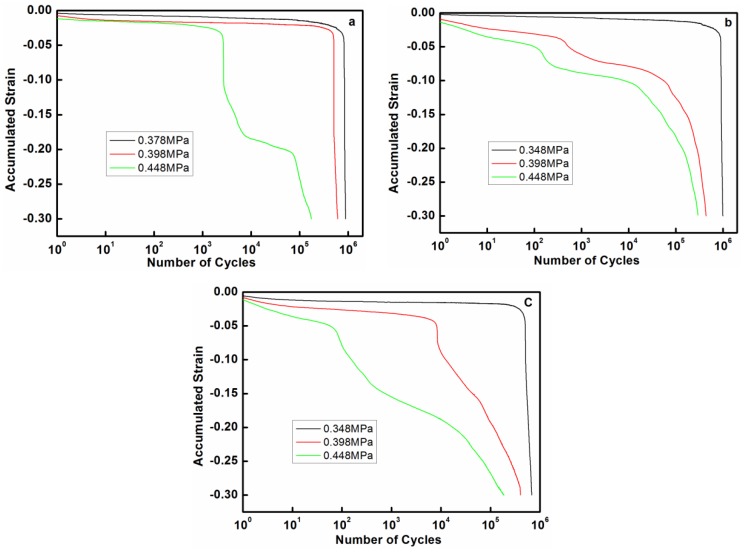
Strain curves of the accumulated strain vs. number of cycles for copper foam: (**a**) 5PPI; (**b**) 20PPI; (**c**) 40PPI.

Figure 2 shows the accumulated strain as a function of cycle numbers for copper foam. Similarly, there is an instantaneous compression strain upon the application of cyclic load. Subsequently, a short duration that starts with the occurrence of the abrupt strain jump. As shown in this figure, an increasing stress amplitude related to a higher plastic instability. Correspondingly, a larger pore size results in longer fatigue life especially at low stress amplitude. In order to analyze the effect of pore size on the fatigue life of copper foam more accurately, microstructure morphologies of failed samples which were tested at low stress amplitude were detected with different instruments.

### 3.2. Microstructure Morphology

**Figure 3:**
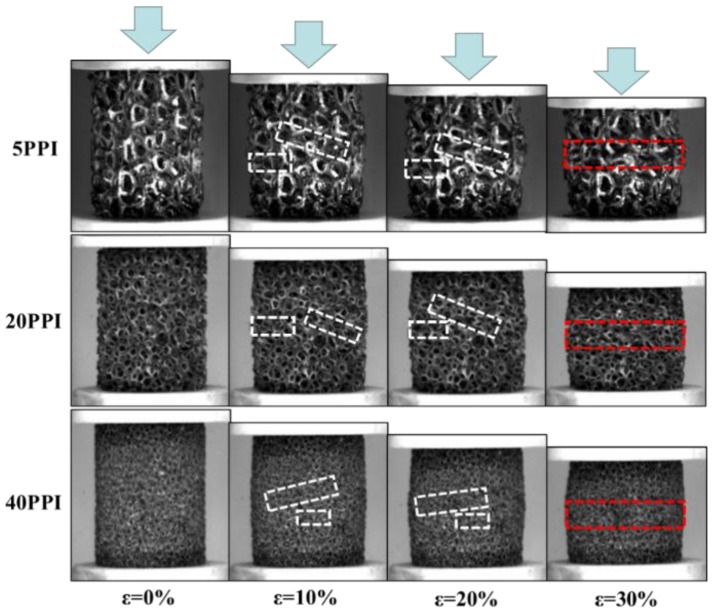
Macroscopic deformation of open-cell copper foam at different stages.

The macroscopic fatigue deformation of specimen at lower stress amplitude were captured by non-contact video extensometer is shown in Figure 3. From this figure, it can be found that the failure plane of copper foam at the initial stage of fatigue deformation is not exactly parallel to the horizontal direction, it can exhibit a certain tilt angle with the horizontal plane. Meanwhile, the deformation area is basically in the middle of the sample, as shown in the white dashed line box in Figure 3 for ε = 10% and ε = 20%. This phenomenon may be caused by the uneven distribution of defects in the material which result in the different compression strength of the internal parts of the material. Most of the pores in the deformed area have undergone plastic deformation, and a few parts have collapsed completely, and the holes are broken down layer by layer under continuous cyclic load. Therefore, it can be seen that the central area of the material is subjected to the worst fatigue damage in the cyclic loading process. Subsequently, the collapse area merges into a large horizontal deformation area in the middle of the specimen with the continuation of fatigue loading, as shown in the red dashed box in Figure 3 for the ε = 30%. Therefore, it can be concluded that the macroscopic deformation characteristics of 5, 20, 40PPI copper foam is similar, little influence can be observed at this scale. Therefore, the effect of pore diameter on micro deformation mechanism of copper foam were further investigated by metallographic microscope and scanning electron microscope. 

**Figure 4:**
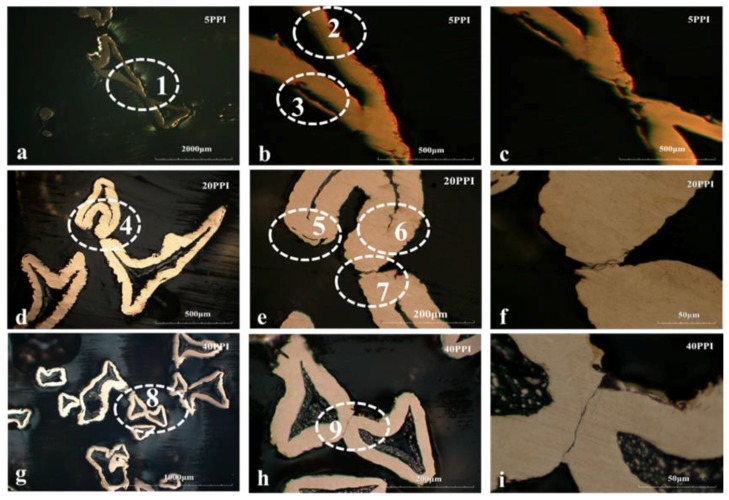
Cross sectional microstructure of copper foam after fatigue failure: (**a**–**c**) 5PPI; (**d**–**f**) 20PPI; (**g**–**i**) 40PPI.

Figure 4 shows the cross sectional morphology of the fatigue-failed sample at lower stress amplitude which was polished after encapsulation by epoxy resin. As shown in Figure 4, copper foam with different pore size related to different fatigue deformation behavior. Figure 4a–c shows the cross section of 5PPI copper foam compressed to 30% deformation at 0.378 MPa stress amplitude. It is clear that the cracks marked by the white dashed line box initiate in the inner side of strut (as shown in Figure 4b) as well as several cracks occurred at the joint of strut (as shown in Figure 4c). Figure 4d–f shows the cross section of 20PPI copper foam compressed to 30% deformation at 0.348 MPa stress amplitude. Figure 4e shows that cracks occurred in the inner side of the strut similarly as well as several cracks can also be detected at the sharp corner of the edge and the joint of strut simultaneously. Figure 4g–i shows the cross section of 40PPI copper foam compressed to 30% deformation at 0.348 MPa stress amplitude. Specially, cracks only can be detected at the joint of strut, as shown in Figure 4i. In comparison, there are several cracks on the strut of sample 1# and sample 2# but no crack on sample 3#, which indicate that larger pore size lead to longer cycle fatigue life at low stress amplitude by reducing the stress concentration effect.

**Figure 5:**
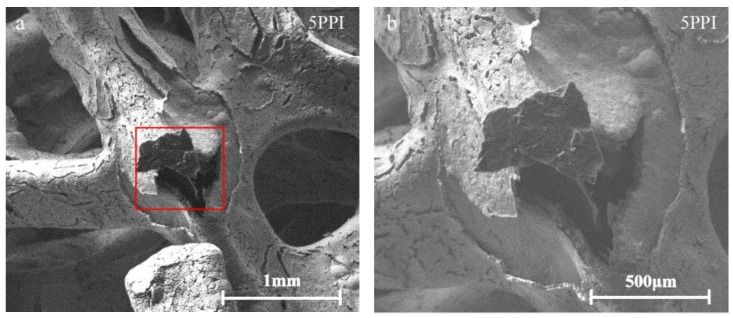

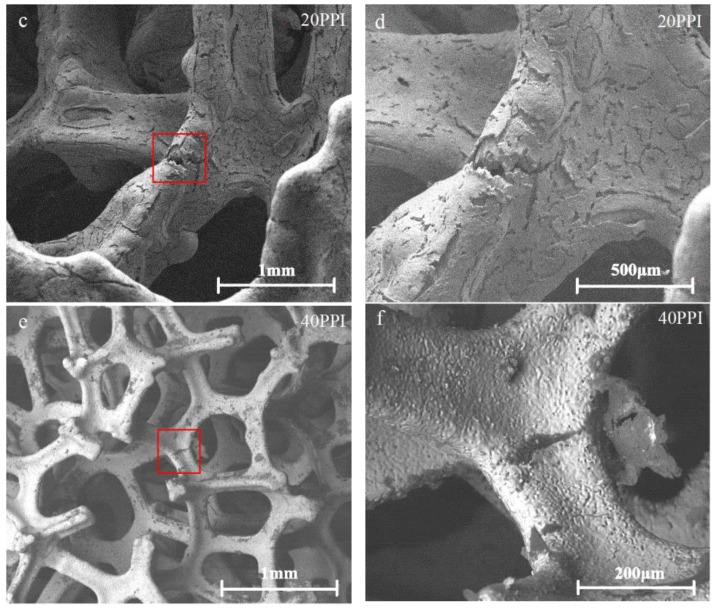
Surface morphology of copper foam after fatigue failure: (**a**,**b**) 5PPI; (**c**,**d**) 20PPI; (**e**,**f**) 40PPI.

The surface morphology of open-cell copper foam after fatigue failure are shown in Figure 5. 5PPI copper foam was tested at 0.378 MPa stress amplitude while 20 and 40PPI copper foam were tested at 0.348 MPa. Figure 5a,b show that large cracks occurred at the joint of strut as well as the shedding of the surface of struts, similar morphology can be detected in Figure 5c,d. However, shedding of the struts can’t be observed but several cracks occurred at the joint of strut when 40PPI copper foam was failed, as shown in Figure 5e,f, which show a good accordance with the cross sectional microstructure of copper foam.

Most of the hollow struts are under the bending loading during the fatigue test and the bend of the hollow struts result in the initiation and propagation of cracks. It has been accepted that the stress at struts is higher than that at the nodes [25]. Stress concentrations were more likely to form at the defects in the hollow struts which originated from the manufacturing process.

**Figure 6:**
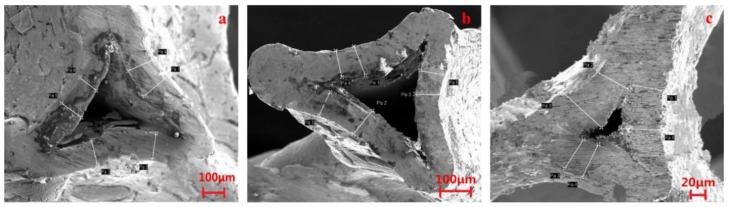
Cross sectional SEM morphology of copper foam: (**a**) 5PPI; (**b**) 20PPI; (**c**) 40PPI.

The typical cross sectional SEM morphology of as-received copper foam are shown in Figure 6a–c. For each sample, 5~7 images were measured. The selected corresponding measurement data of strut are shown in Table 3, they can represent the whole sample very well. The measured thicknesses of the strut of 5PPI, 20PPI, and 40PPI open-cell copper foam were 123.25 μm, 94.67 μm and 73.23 μm, respectively. Comparing the fatigue life of copper foams with three different pore sizes at low stress amplitudes, it can be concluded that a larger pore diameter related to a thicker strut, which induce a better pressure resistance of struts and their junctions.

Combining the morphology characteristics of three different samples, it can be preliminarily confirmed that the bend of the hollow struts results in the initiation and propagation of cracks and on the joints of strut which cause a crucial destruction to sample which accelerate the fatigue failure of specimen. The effect of pore size on fatigue failure characteristics of open-cell copper foam is elaborated.

According to the previous analysis, it can be concluded that 5PPI copper foam have a better compression strength than others at low stress amplitude. In the meantime, the joints of strut have a better anti-pressure ability than struts, cracks are hard to occur on it over a long fatigue cycle. In the process, cracks initiated in the inner of struts firstly which result in the exfoliation of it. However, the destruction caused by the exfoliation of strut is inadequate to cause the large plastic deformation of specimen. With the increase of fatigue cycles, cracks occurs at the junction of the hole during the long-term fatigue damage cause the plastic collapse of hole, which show a good accordance with the microstructure shown in Figure 4 and Figure 6. Subsequently, stress concentrations occur at the adjacent hole and the above-mentioned fatigue failure process was repeated. Comparing with 5PPI copper foam, 40PPI copper foam has a similar macroscopic deformation characteristics but different micro deformation mechanism. Thinner struts and greater density of holes lead to a worse anti-pressure ability of 40PPI copper foam, as more defects are distributed on the nodes where cracks tend to form on it rather than strut. Cracks located at nodes which result in the plastic collapse of holes reduce the anti-pressure ability of specimen significantly. 

As mentioned above, copper foam undergo a local stress concentration in pressure-pressure fatigue test which result in fatigue failure eventually. In the work, the open-cell copper foam with different pore size tend to express diverse fatigue properties.

## 4. Conclusions

This study concerned an analysis the fatigue deformation mechanism of open-cell copper foam at low stress amplitude. A summary of the key conclusions are:

(1) The fatigue failure mechanism of the open-cell copper foam at low stress amplitude is layer-by-layer collapse. Fatigue damage tend to occur at the defects at the initial stage and the deformed region will merges into a large horizontal deformation area in the middle of the specimen with the increase of fatigue cycles.
(2) An increasing in pore size which is related to a thicker strut will result in a better compression strength as well as longer fatigue life when the specimen was tested at low stress amplitude. The strut thickness (correlated to pore size) is the primary factor affecting the fatigue deformation mechanism which is decisive to the fatigue life of the open-cell copper foam. The scanning electron microscopy results are accordant with the experimental data as smaller pore size is correlated with thinner strut and more defects. Stress concentrations can be easily formed in the weak node and result in the initiation of cracks at the joints of strut which cause a severe damage to specimens by accelerating the collapse of holes.

## Figures and Tables

**Table 1:** Results of porosity measurements of the open-cell copper foams.

Sample	Porosity (%)	Pore per Inch (PPI)	Relative Density (%)
1#	93.6	5	6.4
2#	93.7	20	6.3
3#	93.5	40	6.5

**Table 2:** Fatigue test parameters for samples with different pore size.

Samples	Porosity (%)	PPI	Frequency	Stress Amplitude (MPa)
1#	93.6	5	1 Hz	0.378 MPa 0.398 MPa 0.448 MPa
2#	93.7	20	1 Hz	0.348 MPa 0.398 MPa 0.448 MPa
3#	93.5	40	1 Hz	0.348 MPa 0.398 MPa 0.448 MPa

**Table 3:** Measurement data of the thickness of strut.

Strut Number	Thickness (μm)		
	5PPI	20PPI	40PPI
Pa 1	144.1	108.8	59.32
Pa 2	115.0	104.8	68.86
Pa 3	118.6	85.21	66.88
Pa 4	140.1	93.86	71.22
Pa 5	120.5	90.30	96.64
Pa 6	101.2	85.03	76.47
Average	123.25	94.67	73.23
